# A school-based randomized controlled trial to improve physical activity among Iranian high school girls

**DOI:** 10.1186/1479-5868-5-18

**Published:** 2008-04-03

**Authors:** Parvaneh Taymoori, Shamsaddin Niknami, Tanya Berry, David Lubans, Fazloalha Ghofranipour, Anoshirvan Kazemnejad

**Affiliations:** 1 Department of Public Health, School of Health, Kurdistan Medical University, Sanandaj, Iran; 2 Department of Health Education, School of Medical Sciences, Tarbiat Modares University, Tehran, Iran; 3 Faculty of Physical Education and Recreation, University of Alberta, Edmonton, Canada; 4 School of Education, Faculty of Education and Arts, University of Newcastle, Newcastle, UK; 5 Department of Biostatistics, School of Medical Sciences, Tarbiat Modares University,Tehran, Iran

## Abstract

**Background:**

Physical activity (PA) rates decline precipitously during the high school years and are consistently lower among adolescent girls than adolescent boys. Due to cultural barriers, this problem might be exacerbated in female Iranian adolescents. However, little intervention research has been conducted to try to increase PA participation rates with this population. Because PA interventions in schools have the potential to reach many children and adolescents, this study reports on PA intervention research conducted in all-female Iranian high schools.

**Methods:**

A randomized controlled trial was conducted to examine the effects of two six-month tailored interventions on potential determinants of PA and PA behavior. Students (N = 161) were randomly allocated to one of three conditions: an intervention based on Pender's Health Promotion model (HP), an intervention based on an integration of the health promotion model and selected constructs from the Transtheoretical model (THP), and a control group (CON). Measures were administered prior to the intervention, at post-intervention and at a six-month follow-up.

**Results:**

Repeated measure ANOVAs showed a significant interaction between group and time for perceived benefits, self efficacy, interpersonal norms, social support, behavioral processes, and PA behavior, indicating that both intervention groups significantly improved across the 24-week intervention, whereas the control group did not. Participants in the THP group showed greater use of counter conditioning and stimulus control at post-intervention and at follow-up. While there were no significant differences in PA between the HP and CON groups at follow-up, a significant difference was still found between the THP and the CON group.

**Conclusion:**

This study provides the first evidence of the effectiveness of a PA intervention based on Pender's HP model combined with selected aspects of the TTM on potential determinants to increase PA among Iranian high school girls.

## Background

Regular physical activity (PA) has a beneficial effect on overall health [1] and is important for combating the escalating problems of obesity and type II diabetes among youth [2]. Despite overwhelming evidence describing the benefits of an active lifestyle, PA decreases with age [3] and adolescents in many Western countries are not active enough to achieve health benefits [4,5]. The activity patterns of adolescent girls are of particular concern because girls' PA activity participation is generally less frequent and of a lower intensity than that of boys [6]. For example, the 2003 National Youth Risk Behavior Survey (US) found that 40% of high school girls compared to 27% of high school boys failed to achieve modest PA recommendations [4]. Concern regarding the PA levels of adolescent girls is not restricted to Western countries. In a study of Iranian adolescents, only 36% of girls (12–17 years) compared to 61.5% boys were at adoption stages of PA indicating that they were achieving PA recommendations [7]. Due to such low reported rates of PA, this cultural group warrants intervention research designed to increase participation in regular PA. Further, female Iranian adolescents face unique cultural challenges that make achieving adequate levels of PA for health benefits even more difficult. Such challenges include few to no expectations that Iranian women do any exercise (even bicycling which precludes many chances for PA from transportation). It should be noted that while it is not illegal for Iranian women to do such activities, it is very much the social norm that they do not and there are definite cultural standards for activity that women can adopt or reject. Therefore, the combination of cultural limitations and physical inactivity in adolescent girls in general make research into effective PA interventions with female Iranian adolescents particularly important.

The physiological and psychosocial changes experienced during the high school years may make adolescence a particularly high-risk period for girls to adopt sedentary habits [8]. However, because children and adolescents spend large amounts of time at school, the school environment can have a powerful influence on their PA behavior [9]. Schools are identified as key institutions for the promotion of PA among youth as they provide access to most of the population and have the necessary facilities and personnel. While break periods such as recess, active transportation to and from school, school sport and the school's physical environment provide opportunities to be active [10], the physical education (PE) curriculum is the central part of the school identified with the promotion of activity [11]. Although interventions to increase PA have been developed in a variety of settings and have used a range of behavioral science theories to guide intervention design, the majority of school-based interventions have evaluated enhanced PE programs in primary school settings [12,13].

**Figure 1:**
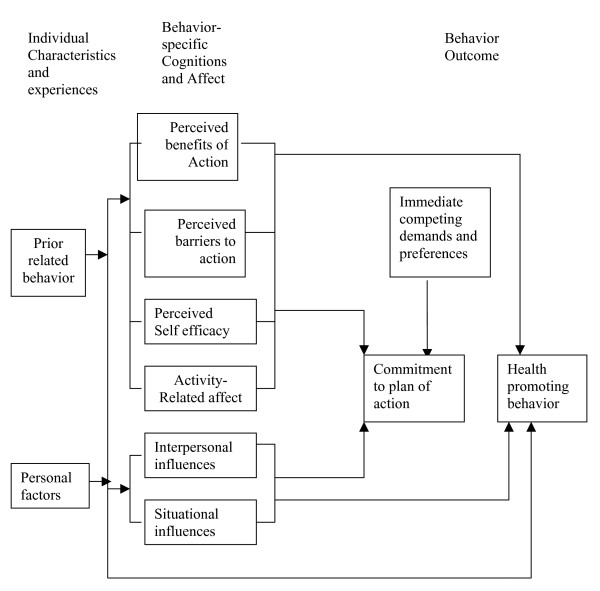
Pender's Health Promotion Model.

Theory-based research is necessary to conduct school-based interventions and one potentially useful theory is Pender's Health Promotion Model (HPM). This model is derived from Bandura's Social Cognitive Theory (SCT) [14] and includes constructs that are proposed to influence health behavior. These constructs include: 1) individual characteristics and experiences (e.g., personal, biological, psychological, and social factors, and prior related behavior); 2) behavior-specific cognitions and affect (e.g., benefits and barriers to action, self-efficacy, interpersonal influences from family, peers, and providers and activity-related affect); and 3) commitment to a plan of action and responses to immediate competing preferences. Taken together, these constructs represent a critical area to assess and target in an intervention directed at motivating individuals to engage in health-promoting behavior (see Fig 1) [15].

Of particular interest to the current research, competing preferences are viewed as alternative behaviors (e.g., watching television) with powerful reinforcing properties over which individuals exert a relatively high level of control. They are last-minute urges based on one's preference hierarchy that can derail a plan of action for positive health action. For example, "giving in" to a competing preference might include watching television or playing computer games rather than being physically active. Individuals vary in their ability to sustain attention and avoid disruption of health behaviors but in general, the inhibition of competing preferences requires the exercise of control capabilities. In the HPM, competing preferences are proposed to directly affect the probability of occurrence of behavior as well as moderating the effect of commitment [15].

Another model that can be applied to this research area is the transtheoretical model of behavior change (TTM), which includes the stages of change that a person moves through when adopting a behavior and the processes of change used in the stages to help in the adoption of a new behavior. The framework proposes that individuals move through a temporal sequence of five stages: pre-contemplation (no intention of becoming regularly physically active), contemplation (intending to become regularly physically active within the next 6 months), preparation (intending to become regularly physically active within the next 30 days), action (being regularly physically active for less than six months), and maintenance (active for more than 6 months) [16]. Marcus and Rossi showed that there are ten processes of change used to move through stages of exercise behavior and that these processes could be categorized as either cognitive or behavioral. In the earlier stages of change, people rely mainly on five cognitive processes whereas in the later stages of change, behavioral processes are more often used [17]. PA researchers have applied the TTM to better understand the adoption and maintenance of exercise behavior change and to apply interventions tailored to stages of motivational readiness [18,19].

As no single theory can account for all complexities associated with behavior change, integration across major theories is recommended in research [20]. Therefore, because the HPM includes individual characteristics and experiences without consideration of environmental influences on PA [15], two TTM behavioral processes that specifically address environmental influences, stimulus control and counterconditioning, were selected for inclusion within one of the intervention groups in this research. Theoretically, these processes of change allow for restructuring of the environment (stimulus control) or for substituting active behaviors for sedentary behaviors (counter-conditioning), thus addressing a gap in the HPM. More specifically, stimulus control includes changing the environment by controlling environmental cues for unhealthy habits while adding stimuli for more healthy options whereas counter conditioning involves changing responses to environmental stimuli and is therefore the process of substituting positive healthy behaviours for unhealthy behaviours [16]. By including these two processes, the intervention targeted both the reaction to environmental stimuli for unhealthy options while also minimizing the occurrence of these stimuli within the environment. Thus, these processes of change were specifically selected to promote participants' control capabilities in order to decrease preferences for sedentary activities with the intention of examining whether these can result in a decline of the effect of competing preferences. A further basis for this addition is that according to Nigg et al. [21] and Burkholder et al. [22], these behavioral processes of change rely on information generated from the environment (i.e., outside of the individual). Research has also shown that counter conditioning appears to be a critical change process for adolescents attempting to change PA behavior [23]. Therefore, these processes targeted an important HPM construct that was specific to participants' TTM stage of behaviour change. Although previous research has integrated HPM and TTM to examine diet in adolescents [24] and in PA with adolescent girls [25], the use of only these two carefully selected TTM constructs and stage of change within the HPM is novel to this study.

Researchers have identified the importance of designing targeted interventions that address the needs of specific populations. Due to cultural barriers that prevent Iranian women from exercising in public places and do not encourage PA in general, research is needed to establish ways to help adolescent Iranian females to develop and sustain active lifestyles. Therefore, the purpose of this study was to evaluate the post-intervention and six month follow-up effects of a tailored PA intervention for Iranian adolescent girls based on the Pender's health promotion model (HPM) and selected constructs from the transtheoretical model (TTM), using a randomized control trial within schools as the medium for the intervention. Three groups were included in the study: an HPM intervention group with HPM interventions addressing stages of behavior change, an HPM with two TTM processes added group (THP), and a control group who received their usual PE program. The main study hypothesis was that the integrated model would result in improved PA maintenance. It was further hypothesized that the THP group would result in greater increases in PA-related cognitions and PA participation compared to the HPM only group.

## Methods

All three groups completed questionnaires prior to the start of the interventions, immediately following the intervention, and six months after the end of the interventions. The research was approved by the Tarbiat Modares University ethics board and the appropriate educational authorities. Prior to participation, investigators sent a written information sheet and consent form for the parents and participants to sign.

**Figure 2:**
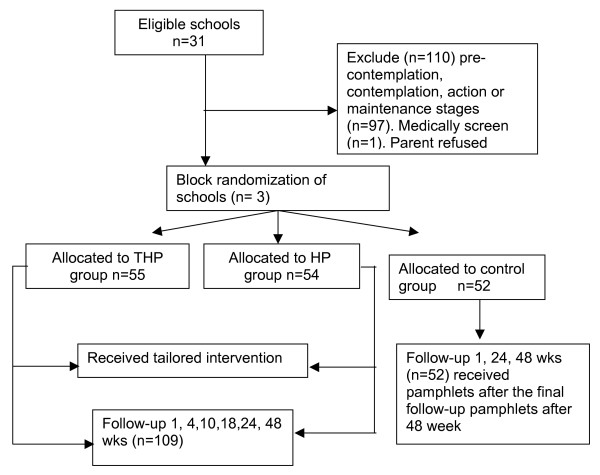
Protocol of the intervention study.

Participants came from three, all female, Iranian, public secondary schools that were randomly selected using a random number table from a possible 31 schools in the area. All selected schools chose to participate. The teachers were all female and all schools were from the same socioeconomic background. Participants within the schools were eligible for inclusion in this study if they were in the preparation stage of exercise behavior change at baseline and were in grades nine or ten (Mean age = 14.79, SD = .44). This inclusion criterion was used because it is participants in the preparation stage who are recommended for recruitment to exercise programs as they are most likely to benefit [26]. Further, no interventions have been previously developed for this population, and to keep the complexity of this study manageable it was decided to initially target interventions at participants in the preparation stage of behavior change because these individuals are the most likely to respond to an intervention as they are already considering PA [16]. Based on these criteria, there were 179 eligible participants. Of these participants, 12 chose not to participate, five did not complete the 6-month follow-up questionnaires, and one could not participate in the study due to physical limitations, leaving 161 participants with complete data. The three schools were randomly allocated to one of the intervention groups: THP intervention (n = 55), HP intervention (n = 54) or control (n = 52). Figure 2 outlines the protocol for school and participant selection.

### Measures

All the instruments were translated into Persian by a bilingual researcher and then validated using standard back translation technique [27] by a native Persian health promotion specialist who was also fluent in English. A native English speaker living in Iran backward translated the questionnaires into Enlish. Five bilingual Iranian health behavior, education, exercise psychology, and instrument development experts were asked to evaluate the pilot instrument for appropriateness and relevance of the items. The instruments were then revised and modified. The questionnaires were pilot tested with 115 participants who were students from randomly selected secondary schools in Iran (57 females and 58 males; age range: 12 to17 years). Revisions in wording and presentation were made based on empirical findings and recommendations from pilot study participants.

Intervention study questionnaires were administered by the researcher to students in their classrooms. The investigator remained in the room during questionnaire administration and answered any questions. For each of the next six consecutive days following questionnaire administration (Saturday through Thursday) each participant completed a child/adolescent/activity log (CAAL) [28].

### Instruments

#### Stage of change

Stage of change was assessed using an adapted two question measure [29]. In the first question, participants were asked whether they believed they did sufficient sports or PA (at least 60 minutes most days per week) on a 2-point scale [yes/no]. Those who answered "yes" were asked to select one of the following two options: 1) I'm currently doing enough, but have been for less than six months (action); or 2) I'm currently doing enough, and I have been for more than 6 months (maintenance). Those who answered "no" were asked to select one of the following three options: 1) I'm not sufficiently physically active, and I have no intention to start (pre-contemplation); 2) I'm not sufficiently physically active, but I intend to start in the next 6 month's (contemplation); or 3) I'm not sufficiently physically active, but I intend to start in the next month (preparation). Test-retest reliability of this staging algorithm was k = .85 [30]. The kappa index of reliability for the stages of change, taken over a 2-week period was .90 (n = 50).

#### Self-efficacy

Perceived self-efficacy was adapted from an existing exercise self-efficacy scale [30]. This scale included eight items (e.g., I could exercise even if I was tired) which were rated on a 4-point Likert scale ranging from 1 (not at all confident) to 4 (very confident). Cronbach's alpha value for the self-efficacy score was .90.

#### Physical Activity

PA was assessed using a modified child/adolescent activity log (CAAL) [28]. The CAAL requires participants to recall the activities they participated in the previous day and the number of minutes of each activity. Minor changes in questions were made for Iranian adolescents. For example, some activities in the CAAL such as ice hockey, and ice/roller skating were inappropriate for Iranian adolescents and replaced with mountaineering, skateboarding, vasat-vasat, khat-khat (ball games), and seven stone (similar to hopscotch). An average daily PA score was obtained by summing the total minutes of all the activities performed by the adolescent. A mean score was derived across the six days. The final version of the CAAL used in this study included 23 items. Reported test-retest reliability coefficients for the CAAL ranged from .73 to .94 [28]. When the CAAL was pilot tested with Iranian adolescents the test-retest reliability coefficient was .98.

The discriminant validity of the log in this study was supported by the stages of change. As expected, those adolescents who were in later stages (action, maintenance) reported more minutes of PA (M = 62.88 min ± 27.05 min) than those in earlier stages (pre-contemplation, contemplation, and preparation; M = 23.32 min ± 9.75 min) (M-W Z = -24.02, P < 0.001). Furthermore, stage of change and total number of minutes and mean of minutes activity per week on the CAAL were significantly correlated (r_s _= .76 to .77, P < 0.001).

#### Perceived benefits/barriers

These were measured by a modified scale (one item was deleted from the original scale based on recommendations from the Iranian experts) which included 18 items measuring both benefits (e.g., A reason I might exercise is because when I exercise I have more energy) and barriers (e.g., I might not exercise if I didn't have enough time). Each item was measured on a 4-point Likert scale ranging from 1 (not at all true) to 4 (very true) [31]. The mean score from each subscale was used in the analyses. The Cronbach's alpha reliability coefficient for the benefits subscale was .83 and for the barriers subscale it was .78.

#### Interpersonal influences

Three subscales developed by Garcia and colleagues [31] were used to measure social support (24 items; e.g., How often does your mother exercise with you?), exposure to models (12 items; e.g., In a usual week, how much do the following people exercise – mother), and interpersonal norms (4 items; e.g., My family expects me to exercise...). All items were rated on a 3-point scale ranging from "never" through "sometimes" to" often". The total score for the social support, interpersonal norms, and exposure to models variables were determined by summing the items for each subscale. Test-retest reliability on social support, interpersonal norms and exposure to models subscale were .84, .75 and .80 respectively. Internal consistency for the separate social support subscales for different family members and peers in this study ranged from .81 to .79.

#### Preferences

PA preferences were measured by a scale based on Pender's instrument for measuring variables in the health promotion model [32]. The 9-item scale contains two choices (A = preferences (e.g., I enjoy playing video games) and B = PA (e.g., I enjoy biking)). The higher the score on the preferences measure, the more likely preferences will interfere with PA. The Cronbach's alpha coefficient on the preferences subscale was .83 for this study.

#### Process of change

Counter conditioning (e.g., When I feel tired, I make myself exercise anyway because I know I will feel better afterward) and stimulus control (e.g., I keep things around my home to remind me of exercising) process of change questions were adapted from a questionnaire developed by Marcus and colleagues [17]. Individuals were asked to recall the frequency of occurrence of each of the 9 items over the past month on a 5-point Likert scale ranging from 1 (never) to 5 (repeatedly). The alpha reliability value for the counter conditioning subscale was .70 and for the stimulus control subscale was .83.

### Interventions

The various components of the interventions, the timeline, constructs targeted and educational methods used are outlined in Table 1. In summary, each participant received four 45 to 60 minute group educational sessions (at baseline, 4th, 10th, and 18th weeks). Although all participants were at preparation at baseline, some participants changed stage over the course of the intervention and so each session included educational components tailored to different stages of change. Therefore, stage of change was assessed at each of these time points so that stage-tailored education and counseling sessions could be delivered. Thus, participants were divided into groups based on their reported stage of change and intervention education sessions took place in groups of 5 to 12 girls. This number of participants allowed for active learning and group discussion. The format of each session included lecture, role playing, slides, reminder cards, PA log planning, and educational pamphlets. The four sessions for both THP and HP intervention groups focused on perceived benefits and barriers to PA and goal setting exercises as outlined in Table 1. Attempts were also made to support the participants' efforts to be active, to develop their support network, and to share their PA goals with their active friends so the friends could help the participants meet PA goals. Although all participants received information in all these areas, participants in maintenance and action stages also received information on how to identify risk factors for future relapse and feedback designed to enhance their self-efficacy. Participants in the preparation stage were given tips on how to start to be active. Participants in the contemplation stage had the benefits and barriers identified which in addition to being aspects of the HPM are also cognitive TTM processes. The educational programs took place within the schools and were conducted by the first author with the help of another female health behavior and education expert who received five hours of training from the first author prior to the start of the interventions.

**Table 1:** Breakdown of specific intervention components by time, targeted construct and methods

Time	Targeted theoretical constructs	Methods used
Baseline	Perceived benefits of physical activity.	Group Educational session targeting: - Specific written information about the benefits of PA and advice on how to make PA a part of their daily lives
	perceived barriers to physical activity	- Individual counseling to provide tailored messages on strategies to overcome perceived barriers (e.g., inclement weather can be a barrier to walking that can be replaced doing exercise videos at home) - Commitment to be physically active was emphasized. - Participants received their log books and written educational materials.
	Interpersonal influences	- In groups, teachers were educated about the importance of perceived social support and modeling so that they provide role modeling by doing exercise in the schools
Week 4	self-efficacy	Group Educational session targeting: - setting realistic goals by learning to start slowly and gradually increase number of days and number of minute of activity per day based on previously agreed goals. - an increase in awareness of small changes that could be made to increase physical activity
	perceived barriers to physical activity	- Individual counseling to provide tailored messages to review personal and environmental barriers and discuss on previously agreed goals. - Participants received a reminder card and pamphlets.
Week 10	perceived barriers to physical activity	Group Educational session targeting: - Help to determine types, frequency, duration and intensity activity they can realistically fit into their daily schedule.
	Interpersonal influences	- Small groups of girls formed to receive peer support, increase exposure modeling and interpersonal norms - Help to develop social network by sharing commitment and plan of exercise. - In groups, the participants' mothers received education about the importance of social support and family PA norms.
	Counter conditioning and stimulus control (only for THP group)	Individually: - Consult to determine when and what type of activity works best with their particular lifestyle and reward themselves for meeting their goals. - Identify and avoid stimuli and other causes that provoke inactivity Group Educational session targeting: - Change surrounding to support goals by posting motivating message. - Substitutions of physical activity for sedentary behavior suggested (e.g., taking fitness break by walking instead of taking a sedentary coffee break). - Participants received a reminder card and pamphlets.
Week 18	perceived PA barriers and self efficacy.	Individually: - Consult to develop a PA plan in which they engage in PA at the recommended levels - Contract renegotiation also deliver positive feedback and verbal persuasion. - assist to identify risk factors and strategies to overcome future relapses such as during vacations, stressful life events, boredom of the PA routine (action and maintenance stages).
	interpersonal influences	In groups, the participants' mothers were educated in the importance of social support and family PA norms.
	Counter conditioning and stimulus control(only for THP group)	Group Educational session targeting: - The avoidance of controlling stimuli and other cases that support inactivity and substitution of physical activity for sedentary behavior. - Participants received a reminder card and pamphlets.
Week 22	social support	Individual phone call focusing on previously agreed goals and encourage reaching goals.
Week 24	interpersonal influences	Group mountaineering

The THP group also received education on the two processes of change: counter conditioning and stimulus control. The counter conditioning education included information on walking or bicycling to school or the store instead of using the bus, getting on or off the bus several blocks away from the destination, taking the stairs instead of the elevator, and taking fitness break by walking or doing desk exercises instead of taking a sedentary coffee break. The stimulus control training included tips on how to change one's environment by posting motivating messages and by removing things in the environment that contribute to inactivity. The control group received no educational or counseling sessions but did receive the educational pamphlets after administration of the final follow-up questionnaires.

In addition to the educational sessions, each participant from the two intervention groups received a 20 to 25 minute individual counseling session based on personal responses to the questionnaire at baseline, and at the fourth, tenth, and eighteenth weeks of the intervention. These counseling sessions helped the participants to set and review personal PA goals, to determine strategies to overcome barriers to PA, and to review her social network. Each participant was also provided with a reminder card of her goals and asked to display the card in a suitable place at home or in her school notebook.

Teachers in the two intervention schools attended sessions aimed at educating them about the intervention models and the importance of social support and modeling. This was done so the teachers could provide role modeling by doing exercise in the schools and could also provide support to help the participants to reach their PA goals. Two 60 minute sessions, one at the 10^th ^week (68% participation) and one at the 18^th ^week (71% participation) of the intervention were held with the participants' mothers to help the mothers understand the benefits of PA, to highlight the importance of social support, and to help them when they tried to help their daughters reach their PA goals. Further, during the 22^nd ^week of the intervention, each participant was telephoned by the researcher to encourage the participant to maintain her PA and to further discuss her PA goals. During the last week of the intervention the participants went mountaineering with their mothers and teachers, a popular means to be active in Iran, to further encourage PA and social support in the participants.

### Data analysis

The analyses were conducted with the Statistical Package for the Social Sciences (SPSS), version 15.0. Variables were assessed for normality of distribution and logarithmic transformations were performed on self-efficacy, social support and mean PA whereas square root transformations were performed on perceived barriers, stimulus control and preferences. Where assumptions of homogeneity of variance and sphericity could not be satisfied, non-parametric tests were used. At post-test and follow-up, stages of readiness to change PA behavior were merged into two categories: pre-action (pre-contemplation, contemplation, preparation) and action (action and maintenance). This decision was made because at follow-up 40 percent of cells had an expected count of less than five. Although there was not a similar limitation at post-intervention, groups were also collapsed at that time so that equivalent analyses could be conducted. Chi-square tests were used to compare these categories between the three groups at post-intervention and follow-up. Progression in the stages through the time points for the three groups was compared using the Friedman test. Changes in outcome variables from baseline through post intervention and follow-up were assessed by a repeated measure ANOVA to determine differences between intervention groups. Where significant interactions were found, follow-up univariate analyses of covariance (ANCOVA) were conducted to reveal main effects. In these analyses the baseline measures were included as the covariate with the follow-up variable as the dependent variable. All post hoc tests were performed with adjustment for multiple comparisons and intervention effect sizes were calculated using Partial η^2^. For all analyses alpha levels were set at *p *< 0.05.

## Results

Demographic characteristics at baseline are summarized in Table 2. There were no significant differences between groups on any of the demographic or outcome measures at baseline. Progression in the stages was used as one of the criteria of intervention success. A statistically significant difference was found between groups for stage progression at posttest, *X*^2 ^(4) = 51.61, *p *= 0.001, and at follow-up, *X*^2^(4) = 20.2, *p *= 0.001. There was a statistically significant increase in the number of participants in both THP and HP groups who progressed through the stages from baseline to follow-up (Friedman *X*^2 ^(2) = 49.6, 2 *p *< .001, and *X*^2 ^(2) = 43.1, *p *= 0 .001), whereas limited progression occurred in the control group (*X*^2 ^(2) = 1.90, *p *= .38). At the six-month follow-up the THP group had a larger percentage of participants in the action stages than the HP group. The percentage of participants in each stage at posttest and follow-up is summarized in Table 3.

**Table 2:** Baseline characteristics of the participants

Characteristics	(THP) group n = 55 Mean (SD)	Control group n = 52 Mean (SD)	(HP) group n = 54 Mean (SD)
Age (years)	14.77(.48)	14.87(.43)	14.74(.42)
Grade	9.35(.50)	9.59(.49)	9.46(.50)

**Table 3:** Participants' stages of change at post intervention and follow-up

	Pre-contemplation n (%)	Contemplation n (%)	Preparation n (%)	Action n (%)	Maintenance n (%)
Post intervention					
(THP) group	0 (0)	4 (7.3)	6(10.9)	32(58.2)	13(23.6)
Control group	0 (0)	10(19.2)	33(63.5)	7(13.5)	2(3.8)
(HP) group	0 (0)	0 (0)	15(27.8)	32(59.3)	7(13)
Follow-up					
(THP) group	2(3.6)	8(14.5)	10(18.2)	28(50.9)	7(12.7)
Control group	0 (0)	8(15.4)	32(61.5)	10(19.2)	2(3.8)
(HP) group	1(1.9)	15(27.8)	7(13.0)	26(48.1)	5(9.3)

### Post-intervention

Table 4 shows the changes in outcome variables across time for each of the three groups. There were significant interaction effects between groups and time for perceived benefits, self-efficacy, interpersonal norms, social support, counter conditioning, stimulus control, overall time spent being active per week and PA (mean minutes per day), indicating that the groups differed across time. Main effects tests for group at post-intervention, with the baseline values as covariate revealed significant differences for counter conditioning, F = 11.97, *p *= .000, η^2 ^= .16, stimulus control, F = 14.82, *p *= .000, η^2 ^= .15, overall minutes PA per week, F = 31.50, *p *= .000, η^2 ^= .29 and mean minutes PA per day, F = 39.94, *p *= .000, η^2 ^= .34. It was noted that the assumption of homogeneity of variance was not met for stimulus control, counter conditioning and mean PA per day. However, equivalent non-parametric analyses revealed similar significant results. Post hoc analyses showed that the differences for counter conditioning and stimulus control were not significant between the THP and HP groups but there were significant differences between the two intervention groups and the control group (*p *= .003 – .006).

**Table 4:** Changes in outcomes variables during baseline through follow-up.

Variables	Time	THP N = 55 Mean (SD)	Control N = 52 Mean (SD	HP N = 54 Mean (SD)	Group × time
Perceived benefit	Pretest	2.58 (.67)^a^	2.73 (.63)^a^	2.88 (.62)^a^	3.01*
	Posttest	3.57 (.40)^b^	3.22 (.46)	3.35 (.73)	
	Follow-up	3.41 (.46)^c^	3.22 (.46)	3.38 (.46)^c^	
Perceived barriers	Pretest	2.89 (.53)^a^	2.94 (.55)^a^	2.72 (.51)^a^	2.40
	Posttest	1.78 (.41)^b^	2.21 (.60)^b^	1.85 (.53)^b^	
	Follow-up	1.97 (.47)^c^	2.45 (.52)	2.01 (.48)^c^	
Perceived self efficacy	Pretest	1.46 (.46)^a^	1.45 (.44)^a^	1.46 (.44)^a^	7.17**
	Posttest	2.61 (.66)^b^	1.83 (.57)^b^	2.45 (.78)^b^	
	Follow-up	1.97 (.70)^c^	1.64 (.54)	2.03 (.65)^c^	
Interpersonal norms	Pretest	4.07 (2.02)^a^	3.46 (1.88)^a^	3.93 (2.16)^a^	4.37*
	Posttest	6.16 (4.40)^b^	4.39 (1.87)	4.83 (2.26)	
	Follow-up	4.80 (1.56)^c^	4.80 (1.56)	5.20 (2.22)^c^	
Exposure to models	Pretest	11.45 (4.15)^a^	10.58 (3.92)	9.91 (4.23)	2.40
	Posttest	14.0 (b4.91)	10.56 (3.74)	11.17 (4.28)	
	Follow-up	12.45 (3.29)	10.25 (4.47)	11.89 (4.58)^c^	
Social support	Pretest	42.73 (7.49)^a^	43.42 (7.71)^a^	42.56 (6.93)^a^	2.52*
	Posttest	48.96 (6.85)	43.73 (6.72)	48.28 (10.33)	
	Follow-up	49.05 (6.78)^c^	46.31 (7.11)	48.41 (8.07)^c^	
Preferences	Pretest	4.81 (2.90)^a^	5.20 (3.07)^a^	4.85 (3.44)^a^	1.69
	Posttest	1.83 (1.45)^b^	4.07 (2.50)	2.80 (2.47)	
	Follow-up	2.56 (2.46)^c^	3.71 (2.66)	3.19 (2.17)^c^	
Stimulus control	Pretest	3.39 (1.01)^a^	3.07 (.98)	3.46 (.83)	3.18*
	Posttest	4.26 (.56)^b^	3.16 (1.01)	3.67 (1.36)	
	Follow-up	3.93 (.70)c	3.30 (.87)	3.90 (.74)^c^	
Counter condition	Pretest	2.97 (.96)^a^	2.64 (.98)	2.99 (.83)	2.46*
	Posttest	3.86 (.86)^b^	2.78 (.78)	3.36 (1.29)	
	Follow-up	3.36 (.73)^c^	2.72 (.85)	3.29 (1.01)	
Mean PA	Pretest	27.16 (12.02)^a^	30.63 (12.29)^a^	28.56 (11.30)^a^	19.14**
	Posttest	75.80 (27.52)^b^	37.26 (20.45)^b^	73.61 (28.73)^b^	
	Follow-up	60.04 (24.87)^c^	46.26 (21.89)	56.79 (27.58)^c^	
Overall time PA	Pretest	140.88 (62.98)^a^	170.33 (77.57)^a^	157.32 (72.02)^a^	15.50**
	Posttest	371.19 (129.63)^b^	195.96 (99.66)^b^	348.08 (139.02)^b^	
	Follow-up	309.96 (134.32)^c^	245.35 (101.30)	285.94 (141.33)^c^	

* *p *< .05 ** 0 < .005 a = significantly different changes between baseline and post intervention *p *< .001 – .03. b = significantly different changes between post intervention and follow-up *p *< .001 – .03. c = significantly different changes between baseline and follow-up *p *< .001 – .04.

### Follow-up

There was no significant difference between the THP and HP groups for overall and mean PA minutes. However, students in both intervention groups reported significantly more PA than students in the control group. Main effects tests by group at follow-up, controlling for the differences in baseline values, found significant differences for counter conditioning, F = 7.83, *p *= .001, stimulus control, F = 14.02, *p *= .04, overall minutes PA per week, F = 4.32, *p *= .01, and mean minutes PA per day, F = 5.0, *p *= .008. At follow-up, results showed no significant differences between the THP and HP groups for any of above outcomes except stimulus control *p *= .02, but the two intervention groups and the control group differed for both behavioral processes (*p *= .000 – .02). No significant differences were found between the HP and control group for overall minutes and mean of PA.

Main effects for time showed that the THP group had a significant increase for stimulus control between baseline and post-intervention (*p *= .000) and a decrease between post-intervention and follow-up (*p *= .004). There was also an increase for stimulus control between baseline and follow-up for the THP and HP groups (*p *= .001 and .004 respectively). Using the same procedure for counter conditioning showed the THP group had a significant increase between baseline, post-intervention (*p *= .000) and follow-up (*p *= .04). However, there was a significant decrease in counter conditioning between post-intervention and follow-up (*p *= .001). Main effects tests for time showed that the two intervention groups had significantly increased their mean PA between baseline and post-intervention (*p *= .000) and also from baseline to follow-up (*p *= .000).

## Discussion

This study was a randomized controlled trial conducted to compare the effectiveness of two individually tailored PA interventions for adolescent Iranian girls. It was hypothesized that an integrated model that included the stages of change and two behavioral processes (counter conditioning and stimulus control) from the TTM in conjunction within the HPM would result in greater increases in PA related cognitions and PA behavior. This integration of models was hypothesized to address the lack of consideration of environmental influences within the HPM and acknowledges the substantial body of research showing that TTM tailored interventions can promote an individual's progression through stages [33-37]. All participants were in the preparation stage of the TTM at baseline (an inclusion criteria for participation). However, following the intervention adolescents in both intervention groups showed greater progression through stages of change and more PA compared to individuals in the control group. This finding is to be expected because many of the educational activities targeted at HPM constructs mirror TTM constructs (e.g., benefits and barriers to exercise, self-efficacy). Further, this finding is similar to previous studies that found that level of PA increased as individuals moved to a higher stage of change [34-37]. Specific findings from the present study showed that at post-test, more individuals in the THP group (81.8%) and the HP group (72.2%) were in the action stages of the TTM than were participants in the control group (17.3%). Further, in support of the study hypothesis, the participants in the THP group also reported greater use of counter conditioning and stimulus control and increased recognition of PA benefits and social support, when compared to the HP and control groups at post-intervention. These findings supports other TTM research findings that showed that the more advanced an individual is in their stage of behavioral change, the more frequently they will use behavioural processes of change [35,38,39].

As already mentioned, participants in the THP group also reported more PA at post intervention than the HP group, although these differences were not statistically significant. At the end of intervention period both the THP and HP groups reported spending about one hour more in activity per day compared to participants in the control group who increased their activity by only six minutes per day compared to baseline. This finding is likely related to the overall advancement into action stages by participants in both intervention groups because a number of other researchers have found that participants in the action stages reported more PA than those are in the pre-action stages [34-37]. While there were no significant differences between the THP and HP groups, or between the HP and control groups for PA at follow-up, a statistically significant difference was found between the THP and control group in daily minutes of PA. This indication of sustained activity in the THP group may be due to the increased use of behavioral processes by these participants. Another explanation for this finding may be that the THP group reported fewer barriers and more social support for PA than the HP and control groups.

Substantial relapses in behavior are generally observed in many intervention studies "when the intensive intervention ends and external supports are withdrawn" [40]. Therefore, it is promising to note that in the present research, despite a small relapse in behaviors following the intervention, a statistically significant intervention effect could still be detected at follow-up. Ward et al. [41] and Haerens et al. [42] found promising results for changing PA behavior when they used changes in the school environment to facilitate PA, whereas we focused on potential determinants of PA. It would appear that the inclusion of behavioral processes from the TTM into the HPM holds promise for PA behavior change among adolescents. These results are encouraging because Clemmens and colleagues found that helping people progress through just one stage can double their changes of successful behavior changes in the near future [43]. Some of the effects of the THP intervention were sustained at the six-month follow-up. Specifically, there were significant positive medium term effects of the intervention on perceived benefits, perceived barriers, self-efficacy, and stimulus control between both intervention and control groups. Similarly, Van Sluijs and colleagues found positive effects due to their intervention on behavioral processes and self-efficacy at a six-month follow-up [44].

A unique aspect of this research was the involvement of the participants' teachers and mothers with the intention of changing cultural norms and providing vicarious learning for the participants through observing important others engaged in PA. As noted in the introduction, the cultural norms for Iranian females to be active are restrictive. Therefore, it may be that by asking the participants' teachers and mothers to encourage the participants to be more active and to express their expectations that the girls be more active, a shift occurred around social norms for the participants within this study. However, it also may be that changes occurred due to peer learning and further research is required to clarify this finding. In general however, this is an extremely encouraging finding that can be used when creating interventions with similar populations.

Another important strength of the current study was the duration of the intervention (24 weeks) and the inclusion of follow-up assessments. To achieve significant changes in deeply entrenched behaviors, interventions among adolescents should last longer than one semester [45]. While previous interventions with adolescents have found statistically significant improvements in PA behavior following shorter interventions [46,47] the long-term sustainability of behavior in these interventions remains untested. Therefore, the results from the current longer study provide a basis for future research and interventions aimed at increasing PA behavior.

## Limitations

There were several limitations of the present study. First, the data were measured by self-report questionnaire which introduces the possibility of biased results. Another limitation is the assessment of the validity of the CAAL and it is recommended that the CAAL be further validated with an objective measure in Iranian adolescents. It should be noted however, that test-retest reliability of the CAAL in this study was .98. Another limitation is the restriction of these interventions to participants in the preparation stage at baseline. Future research should expand these interventions to include participants at other stages of change at baseline. Such research would also necessitate expanding to other processes of change such as consciousness raising for participants in precontemplation.

## Conclusion

Outcome evaluation showed a positive short-term effect for the intervention groups on stage of readiness, potential determinants of PA and on amount of PA, as both intervention groups increased their PA by approximately one hour per day. Although participants in the THP group reported using more behavioral processes than those in the HP group, no significant differences were found between the THP and HP groups for PA at the post intervention. However, the THP group recorded more PA and more positive results on most of the potential determinants compared to the control group and therefore appears to be the stronger intervention. At the six month follow-up, PA levels had decreased from posttest and fewer students in both intervention groups were in the action stages of behavior change. However, the significant differences between the HP and control groups for PA were not present at the six-month follow-up, but some differences between the THP and control groups were still present indicating that this was the stronger intervention.

Iranian girls face many barriers to an active lifestyle, including lack of suitable places to be active, access to facilities and resources, cultural limitations, and the low importance placed on exercising over other activities such as doing homework or home responsibilities. Therefore, access to equipment and facilities and a supportive environment are important strategies for promoting PA among this group of adolescents [48]. Indeed, restructuring the environment to achieve stimulus control and counter conditioning strategies was found to be an effective intervention strategy with this population. These results will allow for future research and interventions for PA not only for female Iranian adolescents but for similar cultural and immigrant groups that have been neglected to date in the PA literature. These results also provide a basis for research using the intensive THP intervention as described in this research with other demographic groups.

## Competing interests

The author(s) declare that they have no competing interests.

## Authors' contributions

PT participated in the design of the study, performed the statistical analysis and drafted the manuscript. TB participated in the statistical analysis, helped to draft the manuscript, and read the paper critically for theoretical content and interpretation of study findings. DL participated in the sequence alignment and helped to draft the manuscript. SN and FG conceived of the study and participated in its design and coordination. All authors read and approved the final manuscript.
